# Mobile Phone Apps for HIV Prevention Among College-Aged Black Women in Atlanta: Mixed Methods Study and User-Centered Prototype

**DOI:** 10.2196/37987

**Published:** 2023-02-23

**Authors:** Naomi Tesema, Dominique Guillaume, Sherilyn Francis, Sudeshna Paul, Rasheeta Chandler

**Affiliations:** 1 Department of Medicine University of Chicago Chicago, IL United States; 2 Center for Infectious Disease and Nursing Innovation Johns Hopkins University Baltimore, MD United States; 3 Nell Hodgson Woodruff School of Nursing Emory University Atlanta, GA United States; 4 School of Interactive Computing Georgia Institute of Technology Atlanta, GA United States

**Keywords:** Black women, HIV prevention, mobile health, mHealth app, mobile technology, reproductive health, women’s health

## Abstract

**Background:**

Black women in college are disproportionately affected by HIV diagnoses. Mobile apps can facilitate the innovative delivery of accurate HIV and sexual and reproductive health information. However, mobile health interventions are severely underused in this population.

**Objective:**

We aimed to quantitatively and qualitatively explore the perspectives of college-aged Black women on using a mobile health app for HIV prevention and sexual and reproductive health. The data obtained from Black women were used to design preliminary mobile app wireframes and features.

**Methods:**

This explanatory, sequential mixed methods study took place from 2019 to 2020 and targeted Black women who were enrolled in college or who had recently graduated from college. Convenience sampling was used during the quantitative phase, followed by purposive sampling in the qualitative phase. A cross-sectional web-based survey evaluating the willingness to use a mobile app for HIV prevention was conducted in the quantitative phase. Descriptive statistics were used for all variables. A separate focus group discussion was conducted with Black women in college to expand on the quantitative results. Focus group discussions explored their perceptions on HIV and health content delivered through a mobile app along with potential features that participants desired within the app. Using the data obtained, we selected the primary features for the app prototype.

**Results:**

In total, we enrolled 34 participants in the survey, with 6 participating in focus group discussions. Over half of the respondents reported a willingness to use an app that contained pre-exposure prophylaxis content. Women who claimed recent sexual activity reported being more likely to use an app feature that would allow them to order an at-home HIV testing kit than their non–sexually active counterparts. The emerging themes from the focus group session were Black women’s health concerns, HIV risk, sources of health information, and preferred app features. The content in our prototype included speaking with a specialist, HIV and pre-exposure prophylaxis information, holistic wellness, and features promoting engagement and retention.

**Conclusions:**

The results of our study guided the design of wireframes for an app prototype targeting HIV prevention in college-aged Black women. The rapid growth of mobile devices in Black communities, coupled with high rates of smartphone ownership among Black youth, makes mobile health interventions a promising strategy for addressing sexual and reproductive health disparities. Participants in our sample were willing to use a culturally appropriate and gender-considerate app for their sexual health needs. Our findings indicate that Black women in college may be excellent candidates for mobile app–based interventions.

## Introduction

Black women account for approximately 61% of new HIV infections among women in the United States [1,2]. Despite the higher rates of HIV among Black women, most HIV prevention efforts have primarily focused on men who have sex with men (MSM). Currently, the Southern United States leads the nation in new HIV diagnoses, with Georgia having one of the highest rates of new HIV infections nationwide. In the Atlanta Metropolitan Area specifically, 70% of new HIV diagnoses occur among Black populations [1]. New HIV diagnoses are particularly high among young Black adults, with data indicating that HIV incidence among Black people aged 25 to 34 years was 5.5% between 2015 and 2019 [3]. Although data exist on HIV incidence rates among Black young adults, there is limited data on HIV incidence rates among young, Black adult women who are enrolled in college. However, current literature has highlighted that while young, Black adult women in college have higher HIV knowledge levels than their White counterparts, their perceived risk of HIV is incongruent with their objective risk counterparts. [4-6].

Owing to the increasing popularity of mobile devices, technology-delivered behavior interventions are being explored to transfer information about the prevention of and care concerns related to HIV and other sexually transmitted infections worldwide [7-10]. In the United States, nearly 100% of young adults aged 18 to 34 years report to using the internet at least occasionally or report owning a smartphone [11]. The adoption of mobile health (mHealth) apps in health care settings is an innovative solution and has generated enthusiasm for its ability to contribute to optimal health outcomes in at-risk communities [12]. However, limited studies have explored the development of HIV prevention interventions specifically for Black women enrolled in college. Furthermore, despite a heightened focus on using mHealth as a strategy for HIV prevention, no studies have explored the potential use of mHealth in HIV risk reduction within this priority community. Given the pervasiveness of mobile phones and associated apps, it may be best to incorporate aspects of these previously developed internet-based and text message–based interventions for delivery via apps. A systematic review of smartphone interventions addressing the HIV continuum of care found that an app’s effectiveness is dependent on its continual use, as many individuals download health-related apps and discontinue using them for a variety of reasons [13]. Therefore, it is important to understand health apps and their possible continued use across smartphone users and how they can best serve the target population. The use of mHealth apps can improve patient experiences, especially to access health information, facilitate convenient physician-patient communication, ensure transparency in medical diagnoses, and improve short-term outcomes [14]. Although there are several smartphone apps aimed at supporting those living with HIV, very few have been developed for Black women and none have focused on Black women in college. There is a need for more research to assess the willingness of college-aged Black women in using mHealth apps for HIV prevention.

Given college-aged Black women’s high risk of acquiring HIV, especially among those who live in the Southern United States, it is imperative to develop innovative approaches to reduce their HIV risk. The focus of this study was to explore how cultural and environmental influences affect college-aged Black women’s sexual health and their willingness to use mHealth apps to meet sexual health needs. The data collected were used to develop a prototype of an HIV prevention mobile app for cisgender Black women attending college in the Atlanta Metropolitan Area. Our goal was to create a product that is usable, understandable, and resonates with the target demographic.

## Methods

### Overview

This study used an explanatory, sequential mixed methods design, in which quantitative and qualitative data were obtained to provide a comprehensive understanding of the factors driving Black women’s willingness to use mobile apps for sexual health and HIV prevention. To understand the intersection of age, race, and gender regarding HIV prevention interventions, particularly among Black women, a voluntary, anonymous survey was conducted among Black women enrolled in college in the Atlanta Metropolitan Area. These data helped us understand their perceptions regarding sexual health and their willingness to use reproductive mHealth apps. In total, 65 responses were gathered and analyzed using descriptive statistics. To complement quantitative data collection, a focus group was conducted with college-aged Black women. Written informed consent was obtained from all participants during both the phases of the study. All data were deidentified, and participants were assigned unique identification numbers. The participants were not compensated for the quantitative phase of the study. Those who participated in the qualitative phase received a US $30 gift card for their participation.

### Ethics Approval

This study was reviewed and approved by the internal review board at Emory University (study number 00002857).

### Theoretical Frameworks Integration

The social cognitive theory of mass communication provides an agentic conceptual framework to analyze the determinants and psychosocial mechanisms through which symbolic communication influences human thought, affect, and action [15]. This theory emphasizes the various pathways in which communications systems operate to influence individuals’ engagement in health behaviors. In the direct pathway, health behavior changes are promoted by informing, enabling, motivating, and guiding participants. In the socially mediated pathway, media influences link participants to social networks and community settings that provide natural incentives and continued personalized guidance for the desired change. Structural interconnectedness provides potential diffusion paths; sociocognitive factors determine what diffuses through those paths.

The Theory of Gender and Power is a model that has been used to examine HIV-related exposures, risk factors, and effective preventive interventions for women [16]. This model describes three structures: (1) the division of labor manifests as economic exposures such as poverty, poor access to health insurance, being uninsured or underinsured, and being unemployed or having a high demand, low control work environment; (2) the division of power manifests as physical exposures, such as having a partner or partners at high risk of HIV acquisition, history of substance abuse, and limited perceived control; and (3) the structure of cathexis, which refers to social norms and affective attachments, manifests as social exposures such as the desire to conceive, and the lack of knowledge of HIV prevention. Three major social structures characterize the gendered relationships between men and women: the division of labor, the division of power, and the structure of cathexis (the process of allocating mental or emotional energy to a person, an object, or an idea). These theories informed how focus groups were conducted and how questions were dispersed in the web-based survey.

### Recruitment and Eligibility Criteria

Participants in the quantitative phase were recruited through convenience sampling, whereas focus group participants were recruited using purposive sampling, in which individuals who completed surveys were recruited to participate in the subsequent qualitative phase. Various recruitment strategies were used in this study. Passive recruitment was conducted by posting recruitment flyers targeting Black women at Emory University, Georgia Institute of Technology, Spelman College, and Georgia State University campuses from September 2019 to February 2020. Furthermore, the recruitment flyers were electronically shared with on-campus groups that had a majority Black membership (eg, Black and African Student Associations). Flyers were additionally shared on social media accounts, such as Facebook, Instagram, and Twitter, and active recruitment was conducted through word of mouth.

Eligible participants met the following criteria: identity as a Black or African American woman, aged 18 to 29 years, own a smartphone, or either currently enrolled or recently graduated <5 years from an undergraduate or graduate or professional program. Participants were not excluded if they selected another race or ethnicity in addition to Black ethnicity. Eligibility was assessed by using an electronic screener questionnaire. Participants who met the inclusion criteria received confirmation after completing the screener.

### Data Collection

#### Quantitative Measures

Survey data contained items measuring sociodemographic sources of health information, and items from the Willingness to Use Mobile Phone Apps for HIV prevention Survey by Goedel et al [17]. This study was initially developed to measure the acceptability of mobile phone apps for HIV prevention among MSM [17]. At the time of this study, there were very few studies on HIV and digital health among Black women compared with that among MSM and transgender populations. Therefore, the research team adapted this survey and contextualized questions about Black women. We obtained authorization from the authors for access and use of the survey codebook, which was adapted for our study through an extensive literature review looking at surveys developed for Black women. Surveys were administered electronically to participants. Sociodemographic information included race, age, sex, education status, and whether the participants owned a smartphone. Frequency in accessing sources of health information was asked, in which participants ranked 10 sources of health information (1=most frequently used; 10=least frequently used). Willingness to use app features (eg, HIV at-home test kits, GPS location, condom ordering services, and pre-exposure prophylaxis [PrEP] resources), willingness to share medical test results with health care providers and current or past partners through the app, willingness to use apps for HIV prevention information, and preferences and attitudes toward PrEP were assessed using a 7-point Likert Scale (eg, 1=strongly disagree; 7=strongly agree). Recent sexual behaviors, recent substance use or abuse, HIV status, and mobile phone use were assessed using multiple-choice questions.

#### Qualitative Measures

Constructs that were explored within the focus group discussion included health topics that were important to college-aged Black women, sources of health information, HIV and PrEP knowledge, HIV risk perception, and mobile app use. The focus groups lasted approximately 90 minutes and were digitally recorded and transcribed verbatim using thematic framework analysis. The sample questions are listed in Textbox 1.

**Sample focus group questions.**

- HIV prevention app usability: If a mobile app was created with HIV prevention content, how likely are you to use it? (range: very likely to not likely at all); Why? What about an app on this topic that would be most appealing? What are your thoughts about notifications to get tested for HIV via an app? Your thoughts on pre-exposure prophylaxis content including initiation and continued use?
- Features of an HIV prevention app: What features need to be included in the app to encourage use? Would social media components be important? What specifically? What reminders help encourage continued use of an app?
- App content: What health topic would you use an app for? What health topics should be included in a mobile app for you?
- App commodities: What sexually related commodities would you feel comfortable ordering from the app? (eg, HIV test kit, condoms, GPS mapping of HIV-testing or pre-exposure prophylaxis clinics who prescribe for women)

### Data Analysis

Descriptive statistics of the study population were calculated using means, SDs, and ranges for continuous variables and frequencies (ie, percentages) for categorical variables. Owing to the smaller sample size, the 7-point Likert Scale responses were dichotomized into binary agree and disagree responses.

Qualitative data were coded and analyzed using MAXQDA (version 2018; VERBI GmbH) to assess for frequent and emerging themes, patterns, and quotes. Multiple coding procedures were conducted in which transcripts were independently coded (NT and SF), followed by a cross-check of coding strategies and interpretation of the data to enhance credibility. Relevant themes and responses were identified, grouped, and compared. Themes were derived from both inductive and deductive codes, and a codebook was developed that included themes based on survey responses. Disagreements among codes were resolved through consensus of the research team [18].

## Results

### Overview

A total of 65 Black women were enrolled in the study; however, only 34 fully completed the survey for the quantitative phase. Most (32/34, 94%) participants identified as Black, and the remaining participants identified as Black with another race. All participants were between the ages of 18 and 25 years and were enrolled or had recently graduated from a collegiate program at the time of the survey. Unprotected vaginal or anal intercourse over the past 3 months was reported by 29% (10/34) of the participants. Almost all (33/34, 97%) participants claimed to be aware of their HIV status, and 74% (25/34 were familiar with PrEP; Table 1). A separate group of 6 Black women participated in the focus group portion of this study.

**Table 1:** Demographics of sample (N=34).

Characteristics		Values, n (%)
**Race and ethnicity^a^**		
	Black or African American	32 (94)
	Hispanic or Latino or Latina	2 (6)
	Middle Eastern	1 (3)
	White or European	1 (3)
**Age (years)**		
	18-25	34 (100)
**Educational status**		
	Enrolled or graduated	34 (100)
**Employment status**		
	Employed	24 (71)
	Unemployed	10 (29)
**Recent sexual behaviors**		
	Unprotected vaginal intercourse	10 (29)
	Unprotected oral intercourse	12 (35)
**HIV status**		
	Negative	33 (97)
	Unanswered	1 (3)
**Heard of PrEP^b^**		
	Yes	25 (74)
	No	9 (26)
**Recent substance use**		
	Marijuana	11 (32)
**History of STI^c^**		
	Chlamydia	2 (6)

^a^The race and ethnicity question allowed users to choose more than one race to include individuals of multiethnicity.

^b^PrEP: pre-exposure prophylaxis.

^c^STI: sexually transmitted infection.

### Willingness to Use an HIV Prevention mHealth App

Most of the participants indicated a willingness to use an HIV prevention mHealth app. Among the features that participants preferred, they indicated acceptability in using an HIV prevention app for access to PrEP content (27/34, 79%), HIV testing reminders (26/34, 76%), HIV testing and PrEP clinic locators (25/34, 74%), and ordering condoms (25/34, 74%). In addition, 68% (23/34) of women stated that they would be willing to share medical test results with their health care providers through features within the mobile app. Participants (15/34, 44%) were less likely to endorse comfort in their partners with access to the app and health information (Table 2).

**Table 2:** Willingness to use sexual health mobile app features (N=34).

Characteristics	Agree, n (%)
I would order an HIV test kit through a mobile app	19 (56)
I would order condoms through a mobile app	25 (74)
I would utilize GPS mapping of HIV-testing and PrEP^a^ clinics that prescribe for women through a mobile app.	25 (74)
I would be willing to use an app with PrEP content.	27 (79)
I would be willing to use an app to remind myself when to get tested for HIV.	26 (76)
I would be willing to share medical test results with present sexual partner(s) via sharing features within a mobile app.	24 (71)
I would be willing to share medical test results with past sexual partners via sharing features within a mobile app.	16 (47)
I would be willing to share medical test results with my health care provider via sharing features within a mobile app.	23 (68)
I would feel comfortable with my partner(s) having immediate access to my STI^b^ or HIV results within an app.	14 (41)

^a^PrEP: pre-exposure prophylaxis.

^b^STI: sexually transmitted infection.

### Black Women’s Health Concerns

Most women cited their top health concerns centered on reproductive health (ie, fertility), weight, and other health indicators related to weight management and obesity (ie, blood pressure, diet, diabetes, or cholesterol), and mental health. The stigma surrounding mental health in the Black community was cited as a health topic that women wished to discuss more:

I think [mental health] is what we should explore because mental health can be attached to anything. It’s really serious.

Ashley

“Mental health doesn’t necessarily mean you’re crazy because I seek help for anxiety, depression, and bipolar, all of that.” Landi is illustrating how language surrounding mental illnesses are often centering the individual, highlighting their lack of control of their mental health as their problem, and out-of-control by calling it “crazy.”

Kiesha

Generalizing certain chronic illnesses as being common to Black women and listing causes as being owing to diet, culture, and upbringing fails to consider the diversity in dietary patterns and lifestyle practices across the Black population. Conversations about the health care system quickly turned into those about experiences with health care providers. Many women in these groups wanted to receive information from certified health care providers, often citing doctors specifically. Nearly all participants commented on the nature of the physician-patient relationship and how better communication, mainly by having physicians better describe their patient’s condition, could improve the relational dynamic. One participant voiced how a visual presentation of conditions could help, or simply using layman’s terms rather than complex medical terms.

Most study participants in this focus group wanted to obtain their health information from a doctor or a qualified health care provider. As avid phone users, being able to connect to qualified health care providers using mobile technology was viewed favorably. Some women stated that information should be delivered more straightforwardly, and that a direct text or phone call would be useful. Most women are cognizant of racism in the health care system, and 1 woman stated how she Googles her symptoms and looks for other sources before visiting her physician so that when she goes to her appointments, she is knowledgeable about potential diagnoses and options for treatment or intervention. Another participant shared how she had been having symptoms of Polycystic Ovary Syndrome for years, and it took a long time for her to be diagnosed. The participant described how she had to advocate for herself and approached doctors with information related to the symptoms she found on the web. She voiced that her difficulties were partly attributed to racism in the health care system and the devaluing of Black women’s health care concerns by health care providers:

I feel like, as a Black woman... if you’re not a black woman physician, I don’t trust you enough to figure it out, to be honest.

Jazzy

The importance of receiving culturally congruent health care from a health care provider who looked like them was emphasized for the relatability and trust it provides. Participants endorsed the benefits of having Black health care providers who were women and viewed Black health care providers as being helpful in overcoming certain health challenges:

I think that [having a Black health care provider] would be very, very helpful because I have no idea of any black women doctors in Atlanta. That would be very helpful.

Gabby

### Obtaining Health Information

Most participants were open to using social media, search engines, and apps to obtain health information, as long as they are factual and backed by health care providers. However, some women hesitated to obtain health information from social media, citing the source’s reputation as a concern.

Kii: “...using social media would help. I like Snapchat. I’ve found a lot of new information just exploring through that.”

Moderator: “With Snapchat is it like quick, dirty information? Nothing too extensive?”

Kii: “They’ll give you the basic thing in the snap and then you can read more. You can scroll to read more if you want to know more about it. I’ve learned a lot through that.”

### PrEP Knowledge

Most women stated that they had heard of PrEP through school newsletters, videos, advertisements, and knowing the people who took it. Some women cited apprehension about taking PrEP because of side effects, unknown effects, and an aversion to taking medicine in general. One woman said that if PrEP was offered for free and was something all women were encouraged to take to prevent acquiring HIV, she would not have a problem taking it. Although most women were knowledgeable about PrEP, their thoughts regarding taking the pill itself were inconsistent:

But I feel like, as for me and my friend group, we wouldn’t take [PrEP]. Because I think, as women, we take so many pills. You would be hesitant if it was just like you take contraception and you take the morning-after pills, and you have to take this pill. It just seems like a lot.

Shay

One woman questioned the efficacy of promoting treatment interventions as opposed to preventive interventions:

So we are going to promote this pill versus just telling you and educating you on it. So it’s like, we’re just going to give you another pill so you can spend more money to take this pill versus knowing the education piece. If we can promote everything else, we can promote...I promise you, you’ve seen breast cancer awareness all year round when they’re collecting money for breast cancer. Why don’t we see that for the education piece for HIV?

Rasheeda

### Risk of Contracting HIV

When asked who they thought was at risk for HIV, the participants cited a variety of demographic groups, including Black youth, Black women, and those who identified as homosexual. Most women stated that anyone who is sexually active is at risk of contracting HIV. The topic of small sexual networks among Black students was also mentioned by the participants as a risk factor for HIV. Some participants who believed Black women to be at risk for HIV explained that Black women were a vulnerable and marginalized group that are generally affected in society, especially regarding access to health care and resources. Most women were aware that the rate of HIV infection was high within the Black community, citing the stigma of HIV within the Black community that discourages open and frank conversations surrounding sex, a severe lack of education regarding sex, and the tendency of Black people to not practice safe sex or wear condoms. However, many participants assumed that the risk of HIV was equal for males and females within the community.

This participant described small sexual networks that Black women experience, which may increase their risk of HIV:

Racially, we tend to want men from our race or women from our race. But black men are more okay with dating women from other races. I feel like they tend to have more partners than we do. So, just in terms of partner contact.

Jazzy

### Favorable HIV App Features

All women had a smartphone and used social media apps such as Facebook, Instagram, and Snapchat. Apps that were deemed as important enough to keep on a phone included transportation apps (Uber and Lyft), a calendar, health apps, a period calendar, or a fertility tracker. Appeals to social media apps were that they contained features that attracted users to check them every day—notifications, opportunities to interact with friends, and a space where one can learn something new or relevant. Women shared how they enjoyed downloading apps that they perceived as fun and engaging. When women were asked about their perspectives regarding using apps to interact with health care providers and share information, they described how the ability to send information directly to providers through texts or chat features appeared to be easier, especially for sensitive health topics, as this takes away from anxiety-provoking face-to-face conversations:

Having a “Speak with a Specialist” or “Call a Physician” feature “I thought it was a very good idea. One reason is that everybody can’t get to a doctor. You can’t always get a doctor on the phone when you call the clinic or the doctor’s office. So I thought that was good.”

Jessie

### Unfavorable HIV App Features

With much concern about privacy and linked accounts, many have raised the point that being able to link an account via Facebook or Gmail might make signing up easier but could make one vulnerable to account hacking that might compromise their privacy. In addition, certain app features, such as avatars, could be considered inappropriate:

A serious note, I think that personally for me, [avatars] are too childish. That would push me away because if we’re talking about something serious, I don’t want to make an avatar of myself and I don’t want to be in a chatroom talking to a whole bunch of people that may potentially have something that I’m concerned about. Because to me, it tends to turn into something more fun than something more serious.

Kezzi

I was just saying the security, because especially like with Facebook and your personal information, I just feel like that’s way too personal or the opportunity that people could screenshot it and send it out. It’s a little bit too much, especially...Like, I would be apprehensive about putting my status, if that was the case. Or even negative, positive, I just think it would kind of deter me away.

Cardice

Overall, the appropriateness of information and features along with security were the biggest concerns and reservations cited by the groups. In addition, advertisements are not viewed as favorable and are distracting. Many women shared that they would be turned off from using the app if they are bombarded with advertisements.

### The Synthesis of Study Results Into a Viable Prototype

The specific aim of this project was to initiate the prototype development of a mobile app targeting vulnerable heterosexual cisgender Black women. Black women in this sample were willing to use a new app for HIV prevention and general sexual health. In addition to HIV and sexual health topics, Black women also described wanting to see content on other aspects of health and wellness. Careful consideration of racial disparities, privacy, and monetization must be undertaken to ensure that the app fully meets the needs of the target audience. Using the qualitative and quantitative data allowed for the creation of the following prototype with Proto.io is shown in Table 3. Using the obtained data, the research team selected primary features for the prototype that were deemed to be favorable by women in our study. These features include the following: speak with a specialist, HIV and PrEP content, holistic wellness, and features promoting engagement and retention. To emphasize cultural competency, we were intentional with the development of each feature. The graphic presentation of the material, including colors, animations portraying Black women, Black providers, and current topics, reflects that the needs and requirements of the app user were considered throughout the design process.

**Table 3:** Prototype screens representing recommended design requirement.

Feature	Data	Prototype feature
Speak with a specialist	In total, 68% (23/34) of respondents are willing to share medical test results with their health care provider via sharing features. One woman stated, “I thought it was a very good idea. One reason is because everybody can’t get to a doctor. You can’t always get a doctor on the phone when you call the clinic or the doctor’s office. So I thought that was good.”	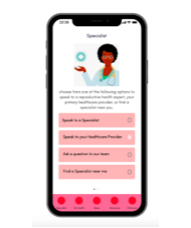
HIV and PrEP^a^ content	In total, 79% (27/34) of survey respondents would be willing to use an app with PrEP content. One participant stated, “...If we can promote everything else, we can promote...I promise you, you’ve seen breast cancer awareness all year round when they’re collecting money for breast cancer. Why don’t we see that for the education piece for HIV?”	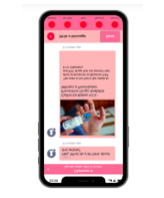
Holistic wellness	“I think holistic wellness should be addressed. Like, ‘Are you drinking enough water? Are you doing what you should be doing? Are you predisposed for this, this or this?’ If it gets your health history and it can give you a window into what could go wrong and what steps you can do to prevent early-onset diabetes or what have you. Just across the spectrum of health and wellness.”	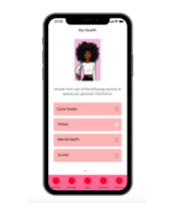
Engagement and retention	Many women use popular social media apps such as Instagram, Facebook, and Snapchat. These apps have features that attract users to check them every day: notifications, opportunities to interact with friends, and space where one can learn something new or relevant. In total, 26% (9/34) of respondents indicated they have not deleted or redownloaded a health-related app in the past 12 months.	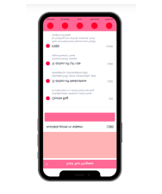

^a^PrEP: pre-exposure prophylaxis.

## Discussion

### Principal Findings

This study examined preferences regarding the use of mobile apps for HIV prevention among a sample of Black women attending college in the United States. Our results provide insights into Black women’s health concerns, perceptions of their health, perceived sexual and reproductive health needs, and willingness to use mHealth apps for HIV prevention. Respondents reported a willingness to use an app containing HIV prevention and PrEP content. There is a scarcity of literature evaluating college-aged Black women’s perspectives on the use of mobile health apps for HIV prevention. Our study demonstrated that college-aged Black women have a high willingness to use mHealth apps for HIV prevention and access information on PrEP. This finding mirrors the results of other studies that have evaluated the use of mHealth to address Black women’s sexual and reproductive health needs, in which Black women have demonstrated relatively high rates of acceptability [19-21]. One study that involved interviewing Black college women about their perceptions toward and knowledge of HIV showed that participants overwhelmingly described using the internet as their primary source of information [22]. These findings suggest that there is ample opportunity to promote digital strategies to support HIV prevention and to endorse positive health outcomes within this key community. Interestingly, the quantitative results indicated that women were less receptive to their partners having access to the app and their health information. This is important to consider as HIV remains a highly sensitive topic, and although digital interventions can be used to facilitate health information sharing, developers must consider whether the inclusion of certain features can threaten end users’ privacy and possibly safety. Prior studies conducted with Black women on the use of mHealth apps have reported similar findings in which end users have voiced concerns about privacy and information sharing [21,23]. Interestingly, although participants were hesitant about their partners having access to an app, they voiced being more receptive to their health care providers having access to their health data through an app. The role health care providers play in reducing HIV risk is critical, and thus opportunities to incorporate communication features with health care providers through digital interfaces should be considered in app development. Incorporating such features within apps that are designed for Black women may aid in reducing the systemic mistrust of health care providers and can be a strategy for engaging this community within the health care system. Although technological advances in mHealth have generated a breadth of opportunities for health promotion research, it also presents numerous challenges, particularly regarding privacy and security. More research must be conducted specifically with college-aged Black women on how to best minimize risks to privacy and security, particularly as HIV and sexual health are sensitive topics that necessitate ethical considerations. Strategies that can be recommended include creating alternate email addresses to log in to the app platform to remain anonymous or using one-time passwords or secure links to verify individuals [24].

The participants in our study identified several features that would encourage mobile app use. Black women spoke of the importance of receiving accurate and culturally competent information from providers who identified as Black women. Similar findings have been reported in other studies, with recent data from the Pew Research Center indicating that Black women aged 18 to 49 years preferred seeing Black health care providers for their routine care [25,26]. Given these findings, an HIV prevention–themed app geared toward this group should include resources that link users to providers to whom they can relate and who look like them. Participants’ responses also suggested a need to develop health promotion apps for college-aged Black women that provide comprehensive HIV and sexual health information so that women can manage their HIV-related risks as precisely as possible. This has been a common finding among women who are Black young adults, who often report not receiving adequate sexual and reproductive health education, including HIV prevention education. Therefore, the content included in app development should focus on providing comprehensive education on HIV, sexual and reproductive health, and other health topics of interest for Black women.

Although our quantitative results demonstrated high rates of acceptability of app use for HIV prevention, our qualitative results suggest that HIV prevention may not be the primary health concern for this community. Black women in our study voiced needing more information on mental health, reproductive health and family planning, and conditions related to weight management and obesity. This finding is crucial, as studies have found that while mHealth apps can optimize and promote health, they tend to be frequently underused after they are downloaded [27,28]. One study reported that the critical drivers of abandonment of mHealth apps include loss of interest, thus indicating the importance of ensuring that apps are developed in a manner that prioritizes the health needs of users [29]. It is plausible that the inclusion of content and features beyond HIV prevention may be key to motivating college-aged Black women to download a potential mHealth app that includes HIV prevention content and addresses their overall health needs. Future research should investigate this finding further to ascertain more data on college-aged Black women’s overall health preferences and the health content they perceive to be important to them. This finding highlights the importance of using human-centered design and collaborative and active engagement with target communities to identify end-user needs and preferences regarding app content and design and must be considered in future research on app development and user engagement [30].

### Limitations

Our study is not without limitations. College-aged Black women in our study were recruited using a convenience sample of Black college students in the Atlanta Metropolitan Area. Therefore, our findings are likely not generalizable to broader populations of Black women of various age groups. The survey that was used in our study was originally developed for MSM populations and was not validated for use among Black women. Although the researchers adapted the survey items toward Black women, measurement bias may have been present, given the lack of validation. Self-report bias could have been present and impacted survey responses and responses from focus group discussions. Our quantitative arm had a high rate of incomplete surveys, possibly related to survey fatigue, which was a limitation of our study. Given the high incompletion rate, significance testing was not conducted because we were not adequately powered to detect a moderate or high effect size. However, our mixed methods study design and thorough investigation of survey responses allowed us to triangulate the quantitative and qualitative data, which was a strength of our study [15,19,22,31,32].

### Conclusions

The rapid growth of mobile devices in Black communities coupled with high rates of smartphone ownership among Black youth makes mHealth interventions a promising strategy for addressing sexual and reproductive health disparities that women who are Black young adults face, particularly regarding HIV. The app prototype was developed for Black women by an all-Black research team to reflect the needs and priorities of this demographic group. Our study corroborates the data we collected and takes a human-centered design approach for the prototype. Given the overall favorable response toward using an mHealth app for HIV prevention, a broader theory-based mobile phone pilot study is warranted to study the impact of apps on the commitment to prevention behaviors among Black women. It is plausible that the app features desired by college-aged Black women in Atlanta overlap with the preferences of women who are Black young adults in different geographical locations. Therefore, conducting a more methodologically rigorous study with a larger group of participants can provide more insight into the best approaches for the development and design of an HIV prevention mobile app tailored for Black women that considers their health needs and preferences. In addition, it will be essential for future studies to collect data on college-aged Black women’s health needs and how to effectively promote holistic wellness within this community while also promoting engagement in HIV prevention behaviors and interventions.

## Abbreviations

mHealth: mobile health
MSM: men who have sex with men
PrEP: pre-exposure prophylaxis

## Data Availability

The data sets generated during this study and analyzed in this study are available from the corresponding author upon reasonable request.
